# Neutrophil extracellular trap-related mechanisms in acne vulgaris inspire a novel treatment strategy with adipose-derived stem cells

**DOI:** 10.1038/s41598-024-51931-w

**Published:** 2024-01-17

**Authors:** Honghao Yu, Boyu Zhang, Yuanyuan Zhan, Yi Yi, Qiong Jiang, Qi Zhang, Yiping Wu, Min Wu

**Affiliations:** 1 Department of Plastic and Cosmetic Surgery, Tongji Hospital, Tongji Medical College, Huazhong University of Science and Technology, 1095 Jiefang Avenue, Wuhan, 430030 Hubei China; 2 Department of Pharmacy, Xianning Central Hospital, The First Affiliated Hospital of Hubei University of Science and Technology, Xianning, 437000 Hubei China

**Keywords:** Mesenchymal stem cells, Infectious diseases

## Abstract

Acne vulgaris is a type of chronic skin disorder caused by *Propionibacterium acnes* (*P. acnes*). Neutrophil extrinsic traps (NETs) play key role in many types of inflammatory skin diseases. Adipose-derived stem cells (ADSCs) was reported modulate immune responses and neutrophil activity. Here, we explored the potential role of ADSCs and the potential mechanism associated with neutrophil extracellular traps (NETs) in relieving acne vulgaris. In the *P. acnes-*infected ear skin model, histological staining was used to evaluate the inflammatory infiltration and NET formation in control, *P. acnes*, and *P. acnes* + ADSCs groups. Besides, western blot was used to detect the expression levels of cit-H3, MPO, and Nrf2 in ear tissue. In vitro, the immunofluorescence staining of MPO and cit-H3, and SYTOX green staining were performed to measure the NET formation. CCK-8 assay, EdU staining, and wound healing assay were used to detect the proliferation and migration abilities of keratinocytes. ELISA assay was utilized to detect the secretion of inflammatory cytokines. In *P. acnes*-infected ear skin, ADSC treatment significantly attenuated inflammation and NET formation via activating Nrf2 signaling pathway. In vitro, the conditioned medium of ADSCs reduced the formation of *P. acne*-induced NETs. Besides, ADSCs could inhibit that the NETs efficiently promoted the proliferation, migration, and inflammatory cytokine secretion of keratinocytes. Our study suggested that ADSCs could attenuate *P. acne*-related inflammation by inhibiting NET formation. This study provides a novel therapeutic perspective of ADSCs in combating acne vulgaris.

## Introduction

Acne vulgaris is a type of chronic skin disorder characterized by inflammation caused by *Propionibacterium acnes* (*P. acnes*) colonizing the hair follicles^1^. Acne vulgaris affects about 80–85% of teenagers worldwide, and also poses serious public health and economic burden due to its apparent sequelae, including scarring, hyperpigmentation, and potential psychological crisis^2^. Early colonization with *P. acnes* and *P. acne*-induced inflammation plays a crucial role in the development of acne vulgaris^3^. Therefore, inhibition of *P. acnes*-induced inflammation is essential for the treatment of acne vulgaris.

Neutrophils could form and release neutrophil extrinsic traps (NETs) after exposure to certain microorganisms or sterile stimuli^4^. NETs are extracellular meshworks composed of nucleic acids and granular or cytosolic proteins. During NET formation, various histones are citrullinated by peptidyl arginine deiminase 4 (PAD-4). Enhanced NET formation has been found in a variety of inflammatory skin diseases, including hidradenitis suppurativa (HS), pyogenic arthritis, pyoderma gangrenosum and acne (PAPA) syndrome, and psoriasis^5–7^. Besides, NETs contribute to skin immune dysregulation and promote inflammation^8^. Acne vulgaris has a prominent neutrophil infiltration at the early stage^9^. Notably, *P. acnes* secret lipases to hydrolyze triglycerides in the sebum to free fatty acids and recruit neutrophils^10^. Undoubtedly, neutrophils participate in the pathogenesis and development of acne to a certain extent. However, the exact role and mechanism of neutrophils, especially NETs, in acne vulgaris remain unclear.

Isotretinoin and antibiotics are commonly used for the treatment of acne vulgaris, but drug resistance and serious adverse reactions are distinguishable factors for poor long-term treatment outcomes^11^. Adipose-derived stem cells (ADSCs) are adipose tissue-derived stem cells with multidirectional differentiation potential and multiple paracrine effects. Especially, ADSCs are widely considered for tissue repair and inflammatory disease treatment, due to their abundant sources, easy availability, and properties such as modulation of the immune response through the release of factors^12^. At present, only a few studies have tentatively demonstrated a therapeutic effect of ADSCs on acne vulgaris, but the underlying mechanisms are not well established. We also noted that ADSC could reduce NET formation and accelerate the clearance of neutrophils in corneal wound healing^13^. Therefore, these pieces of evidence motivate us to explore the potential role of ADSCs in relieving acne vulgaris and the potential mechanism associated with NETs. We used histological staining, western blot, SYTOX green staining, cell counting kit-8 (CCK-8), 5-Ethynyl-20-Deoxyuridine (EdU), and enzyme-linked immunosorbent assay (ELISA) to evaluate the protective role of ADSCs on the inflammatory infiltration and NET formation both in the *P. acne* skin infection mouse model and in vitro model. This study will provide a novel therapeutic perspective of ADSCs in combating acne vulgaris.

## Materials and methods

### Mouse ADSCs isolation and culture

ADSCs were isolated from subcutaneous adipose tissues in the inguinal region of BALB/c mice (male, 6–8 weeks). Adipose tissues were washed with phosphate-buffered saline (PBS) three times, and the blood vessels were removed. Then, the adipose tissues were cut into small pieces and digested with 0.25% type I collagenase (Sigma, USA) at 37 ℃ for 1 h. The DMEM medium (Gibco, USA) containing 10% fetal bovine serum (FBS, Gibco, USA) was used to terminate the digestion. After centrifugation at 400×*g* for 5 min, the cells at the bottom were seeded in a culture dish with DMEM medium containing 20% FBS. The medium was replaced after 24 h to remove non-adherent cells. DMEM medium containing 10% FBS was used to culture ADSCs after passage. ADSCs from passages 3 to 7 were used for subsequent experiments. Mouse keratinocyte cell line PAM212 was cultured with RPMI Medium 1640 (Gibco, USA) containing 10% FBS (Gibco, USA).

### Mouse ADSCs identification

For identification of ADSCs, ADSC suspensions were incubated with anti-CD29 (APC), anti-CD44 (PC7), anti-CD90 (FITC), anti-CD105 (APC), anti-CD31 (PE), and anti-CD34(APC) antibodies (Becton Dickinson, USA) for 30 min and were analyzed with the FACS Calibur cytometer (Becton Dickinson, USA). Mesenchymal stem cell biomarkers CD29, CD44, CD90, and CD105 were the positive markers of ADSCs. Endothelial marker CD31 and hematopoietic lineage marker CD34 were the negative markers of ADSCs. To identify the differentiation abilities of ADSCs, ADSCs were cultured with adipogenic differentiation medium, osteogenic differentiation medium, and chondrogenic differentiation medium (Cyagen Biosciences, China), according to the manufacturer’s instructions.

### Bacterial strains and bacterial culture

*P. acnes* were purchased from BeNa Culture Collection (Henan, China). *P. acnes* were cultured in Columbia Blood Agar Plate (BeNa Culture Collection, China) under anaerobic conditions at 37 ℃ for 5 to 7 days using anaerobic atmosphere generation bags (Mitsubishi, Japan). For in vivo experiments, *P. acnes* were resuspended in PBS at 2 × 10^6^ CFU/mL. A microplate reader (BioTek Instruments, USA) was used to measure the concentration of the bacterial suspension. The standard curve is plotted by measuring the OD value of the known concentrations at 600 nm and fitting a regression line or curve. The concentration of the bacterial sample can then be determined by comparing its OD value to the standard curve.

### *P. acnes* skin infection mouse model

A total of 27 male BALB/c mice (male, 6 weeks) were randomly subdivided into three groups (n = 9 per group): control, *P. acnes*, and *P. acnes* + ADSCs. The sample size was calculated using G*Power software v.3.0 at alpha 0.05 and with 80% of power^14^. Mice in the control group were subcutaneously injected with 50 µL PBS into the middle of the left and right auricle. Mice in *P. acnes* and *P. acnes* + ADSCs group were injected with 50 μL *P. acne* suspension (2 × 10^6^ CFU/mL in PBS) into the same area. After 24 h, the *P. acnes* + ADSCs group was subcutaneously injected with 50 μL ADSC suspension (10^8^/mL in PBS) into the middle of the auricle. The specific concentrations of *P. acnes* and ADSC were determined on the basis of previous studies^15,16^. For comparison, the control and *P. acnes* groups were injected with 50 μL PBS. Mice’s ear thickness was measured with a vernier caliper, and the images of the mice’s auricle middle were photographed daily. After treatment for 48 h, the mice were euthanized with CO_2_ gas, and the ear tissues were obtained for Hematoxylin–Eosin (H&E) and immunofluorescence (IF) staining. The choice of *P. acnes* induction time and the ADSC treatment time was based on previous research and our preliminary experiments^17,18^. This animal experiment was approved by the ethical committee of Tongji Medical College (2022 IACUC Number: 2893) and was performed in accordance with the relevant guidelines and regulations. Besides, the animal experiment followed the recommendations in the ARRIVE guidelines.

### Histological staining

The middle of the auricle were sectioned, and the interval between sections is about 5 μm. For H&E staining, the sections were fixed with 4% paraformaldehyde (Biosharp, China) and embedded in paraffin. Then, the tissue sections were stained with hematoxylin and eosin. The images were obtained with a bright microscope (SDPTOP CX40, China). For IF staining, the tissue sections and cell samples were fixed with 4% paraformaldehyde. The samples were sealed with 5% bovine serum albumin (BSA) and incubated with primary antibody at 4 °C for 12 h. After washing with PBST three times, the samples were incubated with secondary antibodies for 1 h at room temperature. Finally, DAPI nuclear dye was used to stain the nuclei. Anti-Ly6G (1:80, Proteintech, USA), Anti-MPO (1:50, Proteintech, USA), anti-citH3 (1:100, Abcam, USA), and anti-Nrf2 (1:100, Proteintech, USA) antibodies were used as the primary antibodies. Image J was used to select ROIs representing individual cells and the tissue regions of interest. Then, the average fluorescence intensity of the target protein-specific signal and the DAPI signal within the set ROIs were calculated. At least three images of each cell or tissue staining were used for statistical analysis, and the mean and standard deviation were calculated for at least three independent experiments. The relative quantification of the target protein can be calculated using the following formula. The cell fluorescence in control group was normalizated to unity.

$$\text{Cell fluorescence }{\text{of}}\text{ protein} = \frac{(\text{Fluorescence intensity of target protein})}{(\text{Fluorescence intensity of DAPI})} .$$

### Western blot

Total proteins from ear tissue and cell samples were extracted with RIPA buffer (Beyotime, China), and were separated with 10 or 8% sodium dodecyl sulfate–polyacrylamide gel electrophoresis (SDS-PAGE). Then, the separated proteins were electrotransferred to PVDF membranes. After blocking the membranes with 5% BSA for 1 h, the membranes were incubated with primary antibody, including anti-MPO (1:500, Proteintech, USA), anti-citH3 (1:1000, Abcam, USA), anti-Nrf2 (1:2000, Proteintech, USA), anti-GAPDH (1:10,000, Proteintech, USA), anti-IL1β (1:1000, Proteintech, USA), anti-IL6 (1:1000, Proteintech, USA), anti-NF-κB p65 (1:2000, Proteintech, USA), anti-NF-κB p65 (phospho, 1:2000, Proteintech, USA), and anti-β-Actin (1:10,000, Proteintech, USA) antibodies at 4 °C for 12 h, and then incubated with the HRP-linked secondary antibody (1:5000, Proteintech, USA). The blots were cut prior to hybridisation with antibodies. The membranes were next visualized with the enhanced chemiluminescence (ECL) assay kit (Yeasen, USA). Image Lab Software was used to detect and quantify the signals of protein bands. The original data of western blot was included in the Supplementary Information file.

### The isolation of mouse bone marrow neutrophils

The mouse bone marrow neutrophils (BMNs) were isolated from the tibia and femur of BALB/c mice (male, 6 weeks). After the mice were anesthetized and euthanized by dislocation, the tibia and femur were separated and the bone marrow cavity was washed by DMEM/F12 medium containing 10% FBS. The BMNs were separated from bone marrow cell suspension with mouse BMN isolation assay kit (Solarbio, China) according to the manufacturer’s instructions. IF staining was used to examine the purity of isolated neutrophils. The purity of neutrophils was > 90%. The obtained BMN was cultured with RPMI Medium 1640 (Gibco, USA) containing 10% FBS (Gibco, USA).

### In vitro NET formation assay and NET collection

The BMN was treated with PBS (100 µL), PMA (2 µM), *P. acnes* (10^6^ CFU/mL in 100 µL PBS), *P. acnes* (10^6^ CFU/mL in 100 µL PBS) + ADSC-CM (conditioned medium of ADSCs), *P. acnes* (10^6^ CFU/mL in 100 µL PBS) + ADSC-CM + ML385 (Nrf2 inhibitor, 5 µM) and for 4 h. The IF staining (anti-MPO and anti-citH3) and SYTOX green (Solarbio, China) staining were performed to measure the formation of NETs. NETs from *P. acnes*, *P. acnes* + ADSC-CM were collected for subsequent experiments. After treatment for 4 h, the cell layer was washed softly with PBS. Then, PBS solution was added to the wash cell with vigorous agitation. The washing PBS was collected and centrifuged for 10 min at 450×*g* at 4 °C. NETs in the supernatant phase were collected and stored at − 80 ℃.

### Assay for proliferation and migration

The proliferation ability of PAM212 cells was measured with CCK-8 assay (Yeason, China) and EdU incorporation assay kit (RiboBio, China). For the CCK-8 assay, 5 × 10^4^ PAM212 cells were seeded into 96-well plates. After the cells were treated for 4 h, the medium was replaced and 10 μL CCK-8 reagent in 90 μL RPMI Medium 1640 was added to incubate cells for 2 h. A microplate reader (BD, USA) was used to read the absorbance at 450 nm. The cell viability was measured by OD value (450 nm).

$$\mathrm{OD\, value }\,(450\text{ nm})={OD}_{test}-{OD}_{bg,}$$

where OD_test_ and OD_bg_ represent the absorbance at 450 nm of the test and background respectively.

For the EdU assay, the cells in 96-well plates were incubated with culture medium containing EdU for 3 h and fixed with 4% paraformaldehyde. After being washed with PBS three times, the cells were incubated in the Apollo reaction cocktail and Hoechst staining solution in sequence. The fluorescence microscope (Olympus, Japan) was used to photograph the cells. The ratio of Edu positive cells to the total number of cells was used to measure the proliferation ability of cells

$$\text{Edu positive cells }\left(\mathrm{\%}\right)=\frac{\text{Number of Edu positive cells} }{\text{Total number of cells} }.$$

The wound healing assay was performed to assess the migration ability of PAM212 cells. When the cells in 12-well plates reached approximately 90% confluence, the cell layers were scratched with a 1 mL pipette tip and washed with PBS three times. Then, the cells were cultured in serum-free DMEM medium for 24 h. The wound image was photograph by a microscope (SOPTOP CX40, China), and the wound widths was measured by Image J software (NIH, USA). The relative percentage of wound closure was calculated with the following equation.

$$\text{The relative percentage of wound closure} \, \left(\mathrm{\%}\right)=\frac{{W}_{0} -{W}_{24}}{{W}_{0} },$$

where W_0_ and W_24_ represent the wound area at 0 h after scratching and at 24 h after scratching.

### ELISA

The secreted cytokines of PAM212 after treatment of *P. acnes-*induced NETs and *P. acnes* + ADSC-induced NETs were detected with interleukin 6 (IL-6), IL-8, chemokine ligand 1 (CXCL1), and CXCL10 ELISA kits (LiankeBio, China) according to the manufacturer’s instructions.

### Statistical analysis

At least three replicates were performed in each group. All the statistical analyses were conducted with GraphPad Prism 9.0 software, and Student’s t-test was used to compare two different groups. For three or more groups, we conduct one-way ANOVA to compare the differences between different groups. If the one-way ANOVA test shows a significant difference, LSD t test was performed to determine which specific groups differ significantly from each other. P-value < 0.05 was regarded as statistically significant.

### Ethics approval and consent to participate

This animal experiment was approved by the ethical committee of Tongji Medical College.Please note we have moved the section ‘Ethical approval and informed consent’ to the end of the methods, as per house style.

## Results

### Identification of ADSCs and *P. acnes*

**Figure 1:**
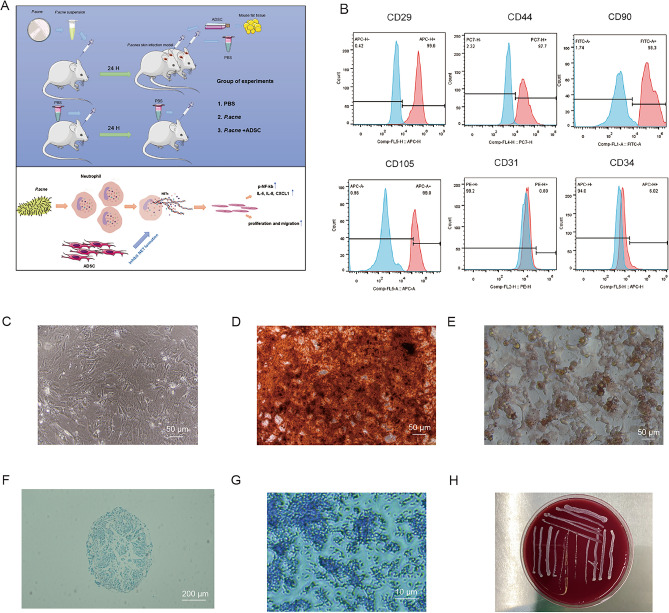
Identification of ADSCs and *P. acnes.* (**A**) Left part, the flow diagram of the in vivo experiment. Right part, the schematic diagram of ADSCs in treating acne vulgaris. (**B**) Flow cytometry analysis of ADSC surface markers, including CD44, CD29, CD90, CD105, CD31, and CD34. (**C**) Morphology of ADSCs at passage 3 (100×). (**D**) Alizarin red staining of ADSC osteogenic differentiation (100×). (**E**) Oil-red O staining of ADSC adipogenic differentiation (100×). (**F**) Alcian blue staining of ADSC chondrogenic differentiation (40×). (**G**) Gram staining of *P. acne* (1000×)*.* (**H**) Digital photographs of the bacterial layer of *P. acne.*

The detailed study process was shown in Fig. 1A. We mainly explored the function of ADSCs and NETs in acne vulgaris. Flow cytometry results showed that positive mesenchymal stem cell biomarkers CD29 (99.6%), CD44 (97.7%), CD90 (98.3%), and CD105 (99.0%) are highly expressed and endothelial marker CD31 (0.8%) and hematopoietic lineage marker CD34 (6.2%) were barely expressed (Fig. 1B). Primary ADSCs were separated from adipose tissue from the groin of mice, which were observed by an inverted microscope showing a spindle-shaped morphology **(**Fig. 1C). Alizarin Red S staining showed red calcium deposition (Fig. 1D). Oil Red O staining results showed small cytoplasmic lipid droplets in differentiated ADSC cytoplasm, which were the features of adipocytes (Fig. 1E). Alcian blue staining showed the formation of blue chondrocyte spheres (Fig. 1F). Surface biomarkers and the abilities of multidirectional differentiation proved that the separated cells were ADSC. Gram staining showed typical colony morphology of gram-positive bacilli (Fig. 1G). In addition, *P. acnes* inoculated on Columbia plates exhibited the normal and energetic bacterial layer (Fig. 1H). These results identified the features of ADSCs and the viability and effectiveness of *P. acnes*.

### ADSCs reduced *P. acnes*-related inflammation and inhibited the infiltration of neutrophils

**Figure 2:**
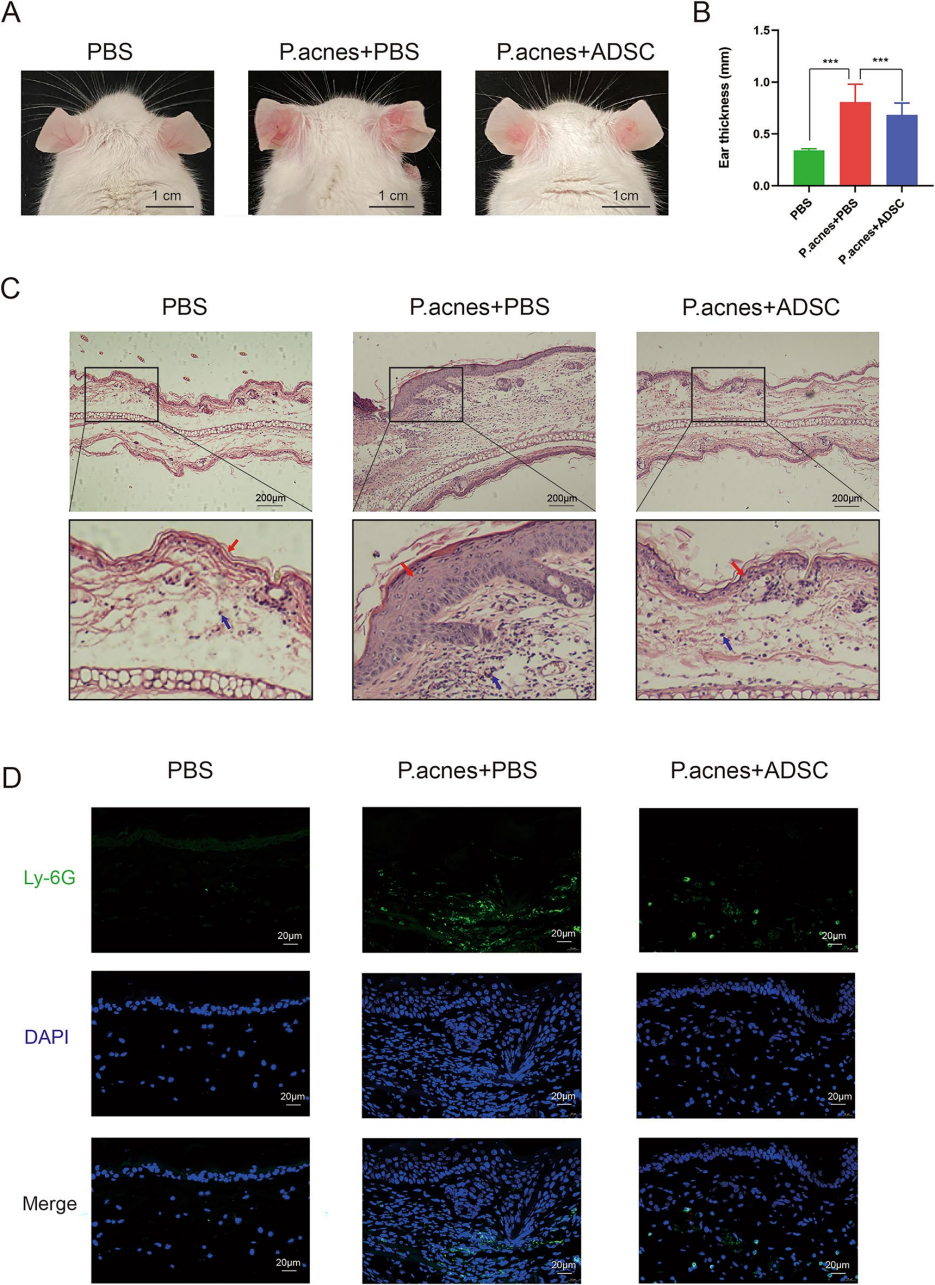
ADSCs reduced *P. acnes*-related inflammation and inhibit the infiltration of neutrophils. (**A**) Digital ear photographs in control, *P. acnes*, and *P. acnes* + ADSCs groups after 24 h treatment. (**B**) Ear thickness measured with a micrometer after 24 h treatment (n = 18). (**C**) H&E staining (40×) of ear tissue sections in each group. Red arrow: stratum corneum. Blue arrow: inflammatory cells. (**D**). IF staining of Ly-6G (green) for ear tissue section (400×). The nuclei were stained with DAPI (blue).

*P. acnes* were subcutaneously injected into the middle of the auricle to establish the *P. acnes* skin infection model. The digital photographs showed that there were significantly less redness and swelling of the auricle in the *P. acnes* + ADSCs group, compared with the *P. acnes* group (Fig. 2A). Besides, the thickness of the middle of the auricle was significantly reduced in the *P. acnes* + ADSCs group (Fig. 2B). H&E staining also showed that infiltration of inflammatory cells and thickening of the skin stratum corneum were attenuated in the *P. acnes* + ADSCs group (Fig. 2C). Ly6G is a specific marker of neutrophils, the number of Ly6G+ cells could reflect the degree of neutrophil infiltration in the tissue. Furthermore, IF staining confirmed that Ly6G+ neutrophils aggregated and infiltrated in the stromal layer after *P. acnes* infection, and ADSC treatment reduced the infiltration of Ly6G+ neutrophils (Fig. 2D). These results suggested that ADSCs significantly inhibited the inflammation and the infiltration of Ly6G+ neutrophils of ear skin in the *P. acnes* infection model.

### ADSCs inhibited NET formation via enhancing Nrf2 signaling pathway in mice

**Figure 3:**
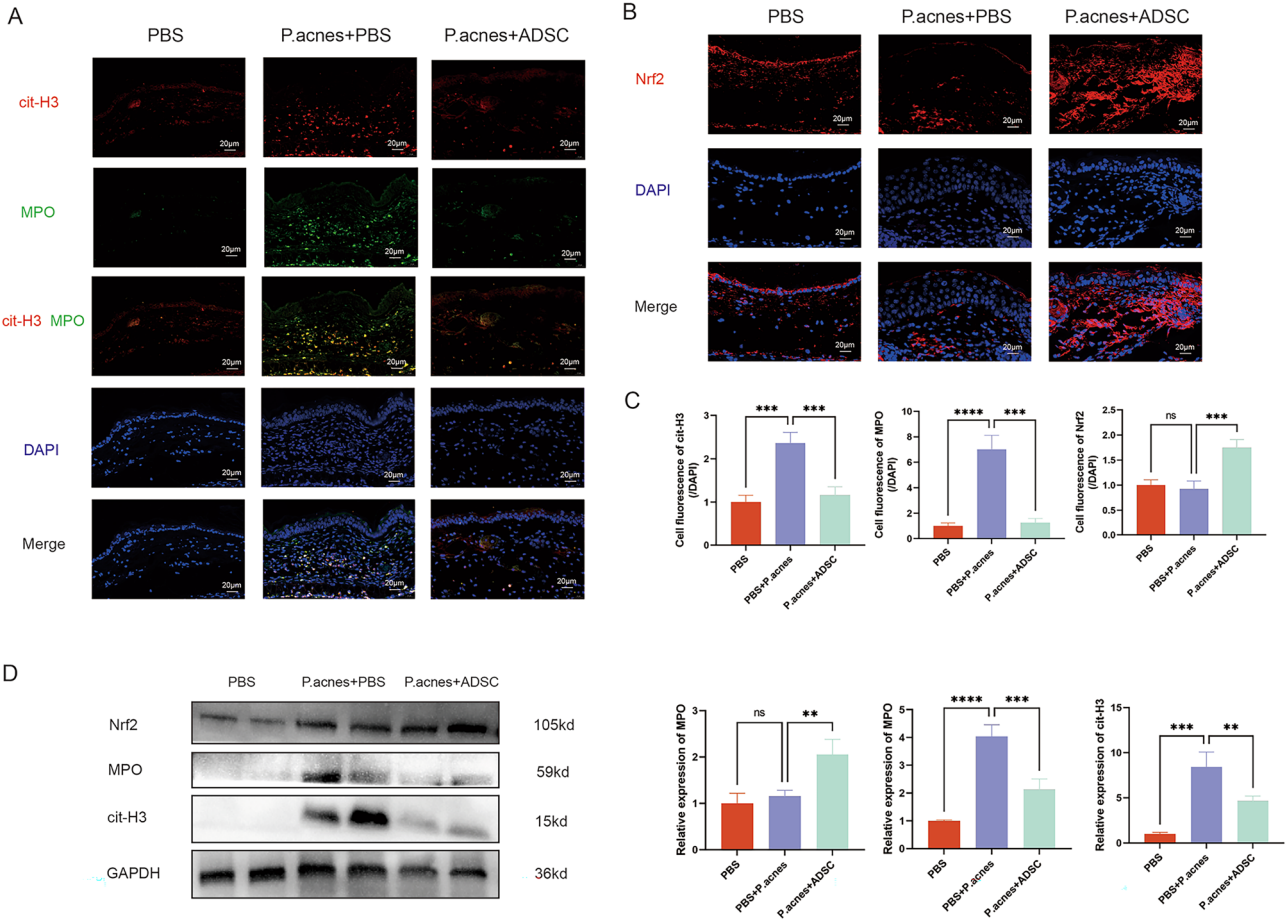
ADSCs inhibited NET formation via enhancing Nrf2 signaling pathway in mice. (**A**) IF staining (400×) of cit-H3 (red) and MPO (green) for ear tissue sections in control, *P. acnes*, and *P. acnes* + ADSCs groups after 24 h treatment. The nuclei were stained with DAPI (blue). (**B**) IF staining (400×) of Nrf2 (red) for ear tissue sections. The nuclei were stained with DAPI (blue). (**C**) Quantitative analysis of IF staining. (**D**) Western blot analysis of cit-H3, MPO, and Nrf2 in ear tissue in control, *P. acnes*, and *P. acnes* + ADSCs groups.

Myeloperoxidase (MPO) and citrullinated histone H3 (cit-H3) are important markers in the formation of NETs^19^. IF analysis revealed that the amount of MPO and cit-H3 were largely increased after infecting with *P. acnes*, but were reduced by ADSCs injection (Fig. 3A). Meanwhile, the expression of Nrf2 was increased in the *P. acnes* + ADSCs group, compared with the *P. acnes* group (Fig. 3B,C). Western blot results also revealed that ADSCs treatment significantly reduced MPO and cit-H3 expression and increased Nrf2 expression in the mouse ear skin (Fig. 3D). This part proved that ADSC treatment suppressed the NET formation of and activated the Nrf2 signaling pathway in *P. acnes*-infected skin.

### ADSCs inhibited NET formation via Nrf2 signaling pathway in vitro

**Figure 4:**
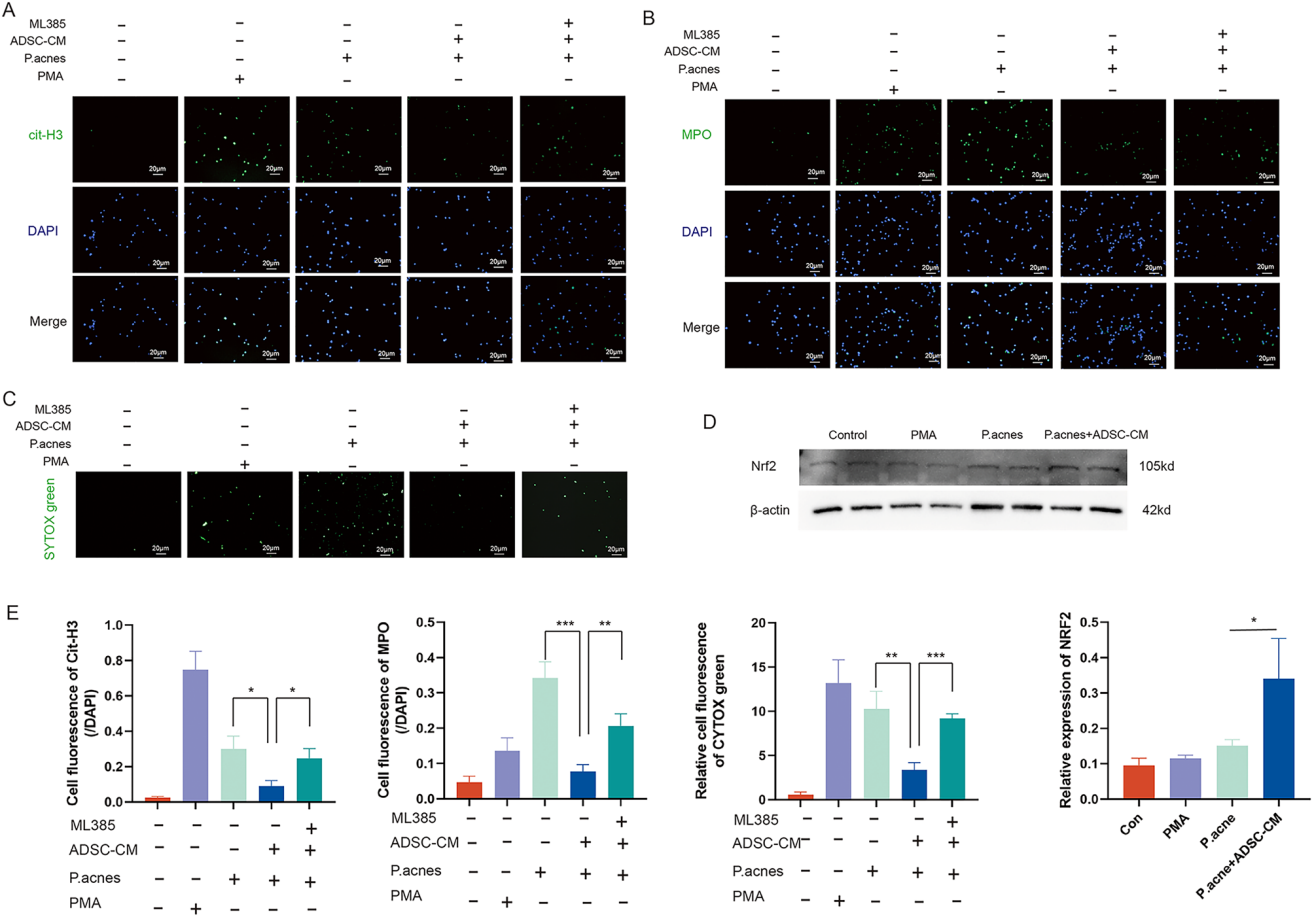
ADSCs inhibited NET formation via Nrf2 signaling pathway in vitro. (**A**) IF staining (400×) of cit-H3 (green) for BMNs in PBS, PMA, *P. acnes*, *P. acnes* + ADSC-CM, and *P. acnes* + ADSC-CM + ML385 groups after 4 h treatment. The nuclei were stained with DAPI (blue). (**B**) IF staining (400×) of MPO for BMNs in 5 groups. The nuclei were stained with DAPI (blue). (**C**) SYTOX green staining (400×) for BMNs in 5 groups. (**D**) Western blot analysis of Nrf2 in BMNs in PBS, PMA, *P. acnes*, and *P. acnes* + ADSC-CM groups. (**E**) Quantitative analysis of IF staining and Western blot.

Next, to decipher the effect of ADSCs on NET formation in vitro, BMNs from the different treatments were prepared for IF staining and SYTOX green staining. The ADSC-conditioned medium (ADSC-CM) treatment reduced the expression of NET markers cit-H3 and MPO, showing that ADSC-CM could inhibit *P. acnes*-induced NETs **(**Fig. 4A,B**)**. Besides, this effect of ADSC-CM could be weakened by a specific Nrf2 inhibitor ML385 **(**Fig. 4A,B**)**. SYTOX green was used to quantify the level of extracellular DNA during NET formation, which demonstrated similar results **(**Fig. 4C**)**. Moreover, western blot showed that the Nrf2 expression of BMN was up-regulated by ADSC-CM **(**Fig. 4D**)**. Therefore, *P. acnes* could stimulate the release of NETs from neutrophils, and ADSC treatment inhibited NET formation via activating the Nrf2 signaling pathway (Fig. 4E).

### ADSCs inhibited NETs-induced proliferation and migration of keratinocytes

**Figure 5:**
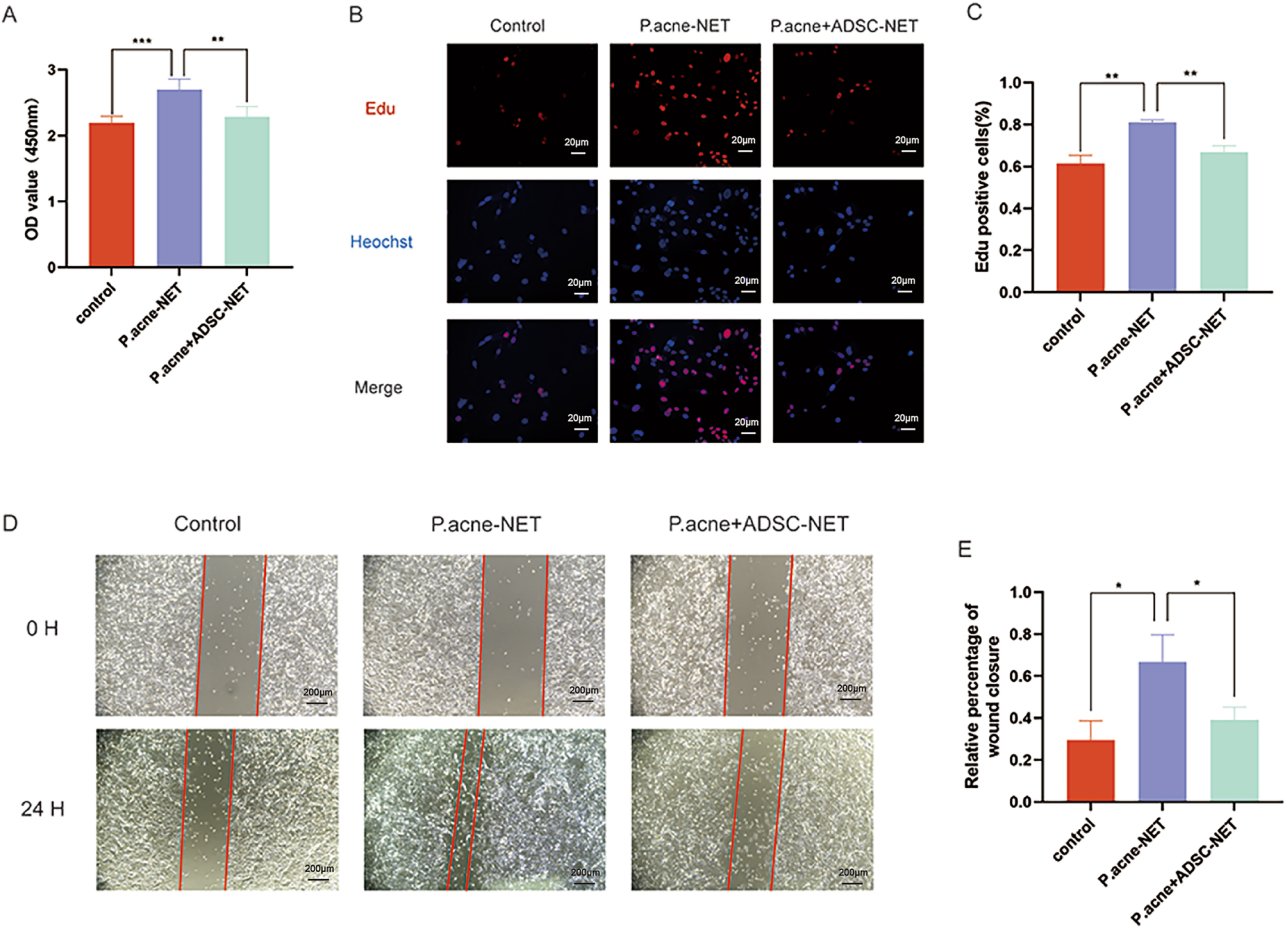
ADSCs inhibited NETs-induced proliferation and migration of keratinocytes. (**A**) CCK-8 assay for PAM212 cells treated with *P. acnes*-NETs (10 μg/mL) and *P. acnes* + ADSC-NETs (10 μg/mL) for 24 h. (**B**) EdU staining (× 400) for PAM212 cells in 3 groups. (**C**) Quantitative analysis of the positive cell percentage in EdU staining. (**D**) Wound healing assay (40×) of PAM212 cells in 3 groups at 0 h and 24 h. (**E**) Quantitative analysis of the wound healing rate for PAM212 cells. *p-value < 0.05, **p-value < 0.01, ***p-value < 0.001.

Thickening of the stratum corneum of the skin is an important histological feature of acne vulgaris. Here, we used *P. acnes-*induced NETs and *P. acnes* + ADSC-induced NETs to treat PMA212 keratinocyte cells. The double time of PAM212 is about 48 h in a complete medium. In CCK-8 assay, *P. acnes-*NETs group exhibited the highest cell viability, while the *P. acnes* + ADSC-NETs group showed inhibited cell viability than *P. acnes-*NETs group (Fig. 5A). Similarly, the EdU assay also showed that *P. acnes* + ADSC-NETs group had markedly decreased EdU + cell number, in comparison to *P. acnes-*NETs group (Fig. 5B,C). These results indicated that *P. acnes*-induced NETs promoted keratinocyte proliferation, while ADSCs treatment could weaken the effect of NETs on keratinocytes. Besides, the scratch assay showed that the wound healing rate of the *P. acnes-*NETs group was significantly more than the other two groups (Fig. 5D,E). This indicated that *P. acnes*-induced NETs promoted the migration of keratinocytes, while ADSCs treatment also weakened the effect.

### ADSCs attenuated NETs-induced secretion of inflammatory cytokines via inhibiting NF-κB signaling pathway

**Figure 6:**
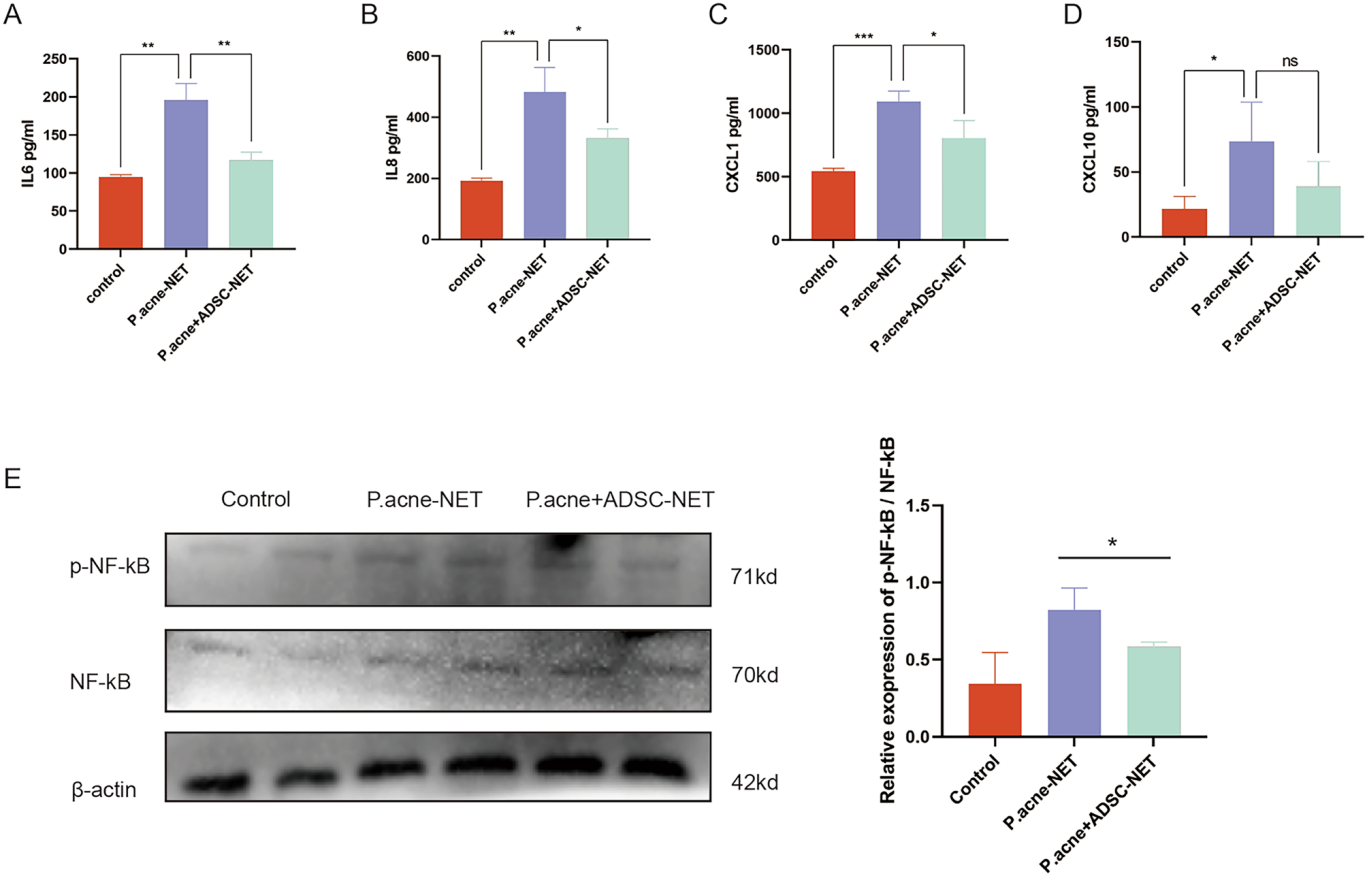
ADSCs attenuated NETs-induced secretion of inflammatory cytokines via inhibiting NF-κB signaling pathway. (**A**–**D**) The IL-6, IL-8, CXCL1, and CXCL10 secretion amounts in PAM212 cell culture supernatants were measured by ELISA. (**E**) Western blot analysis of p-NF-κB in PAM212 cell. *p-value < 0.05, **p-value < 0.01, ***p-value < 0.001.

The release of inflammatory cytokines is a critical participation in *P. acne*-related inflammation. Keratinocytes are one of the main sources of inflammatory cytokines. Here, ELISA assay revealed that IL-6, IL-8, CXCL1, and CXCL10 secretion of PMA212 keratinocytes, were significantly up-regulated in the *P. acnes-*NETs group (Fig. 6A–D). Also, IL-6, IL-8, and CXCL1 secretion were significantly decreased in the *P. acnes-*NETs group compared to *P. acnes-*NETs group. Western blot revealed that p-NF-κB was up-regulated in the NETs group, and was down-regulated in the *P. acnes* + ADSC-NETs group (Fig. 6E). In summary, *P. acnes*-induced NETs promoted the secretion of IL-6, IL-8, CXCL1, and CXCL10 of keratinocytes via NF-κB signaling pathway. It inferred that the inflammation induced by *P. acnes* + ADSC-NETs was significantly attenuated compared to *P. acnes*-induced NETs.

## Discussion

Acne vulgaris is a chronic skin disorder with complex mechanisms. It is intriguing to explore the pathogenesis and novel therapeutic strategies for acne vulgaris. Here, our study revealed that NET formation was increased in the *P. acne* skin infection mouse model, and ADSC treatment significantly attenuated *P. acnes*-induced inflammation and NET formation in ear skin. In vitro, ADSC treatment inhibited NET formation by activating the Nrf2 signaling pathway. Keratinocytes after *P. acnes* + ADSC-NETs treated presented markedly decreased proliferation, migration, and inflammatory cytokine secretion than that of *P. acnes*-induced NETs. Therefore, we preliminarily proved that ADSC treatment inhibited *P. acnes*-induced inflammation via regulating NET formation.

ADSCs play an irreplaceable role in skin damage, repair, and dermatology due to their abundant source, multipotent stemness, and paracrine advantages^20^. At present, ADSCs have been applied in the treatment of rheumatoid arthritis, colitis, and other inflammatory diseases by regulating inflammatory responses^21^. In the field of acne vulgaris, we also noticed just two studies exploring ADSC function. For instance, Zhao et al. demonstrated that the injection of autologous subdermal stromal vascular fraction gel (SVF) into facial areas, significantly attenuated *P. acnes*-related inflammation^22^. Besides, Li et al. demonstrated that the local injection of ADSCs inhibited *P.acnes*-related inflammation by inhibiting IL-1β secretion of macrophages in mouse ears^16^. These two papers mainly elucidate that ADSCs and containing-ADSCs SVF, were able to inhibit acne vulgaris feature by modulating the microenvironmental inflammatory response. Although Li et al. have well reported the anti-acne effect of ADSCs in acne-induced inflammatory response in acne vulgaris, it should be noted that the inflammatory process of acne vulgaris does not involve only macrophages, and the effect of adipose-derived stem cell therapy on neutrophils and keratinocytes in acne vulgaris still needs further research. Our study focused on the effect of ADSC on NET-related inflammation in acne and on the inflammation of keratinocytes, which provided a novel therapeutic perspective of ADSCs in combating acne vulgaris. Our study confirmed that ADSCs treatment also reduced hyperkeratosis and NET formation, indicating that ADSCs might attenuate acne vulgaris via reducing neutrophil-related inflammation and excessive proliferation of keratinocytes. These results provide a new perspective for ADSC treatment of acne vulgaris and validate the anti-acne effect of ADSCs.

NET formation is a key process of neutrophil-induced inflammation, which is closely related to inflammatory and autoimmune diseases and cancers^23^. NETs contribute to the pathomechanism of psoriasis by enhancing IL-17 secretion^24^. Zhu et al. showed that the inhibition of NET formation protected distant organs in the polymicrobial sepsis murine model^25^. Here, our results also revealed that *P. acnes* enhanced the NET formation both in vivo and in vitro. ADSCs had shown anti-inflammatory and antioxidant properties in various other disease models^21^. Nrf2 functions as an intracellular defense mechanism, which exerts antioxidant protection via coordinating the activation of several antioxidative enzymes including superoxide dismutase (SOD), catalase, glutathione peroxidase (GPx), and heme oxygenase-1 (HO-1)^26^. Nrf2 could induce multiple antioxidant enzyme expression to inhibit ROS production and reduce NET-related inflammation. ML385 is a probe molecule that binds to Nrf2 and inhibits its downstream target gene expression. In our study, ADSCs reduced the release of NETs and enhanced the Nrf2 expression. In addition, the inhibition effect of ADSCs on *P. acnes*-induced NETs was weakened when the neutrophils were pretreated with ML385.

Hyperkeratosis and inflammatory cytokine secretion are critical to the pathophysiology of *P. acnes*^27^. We explored the function of NETs on keratinocytes. We found that NETs promoted the proliferation and migration of keratinocytes and the secretion of inflammatory cytokines (IL-6, IL8, CXCL1, and CXCL10) through activating NF-κB signaling pathways. Research has shown that the inhibition of NF-κB signaling pathways reduced IL-1β, IL-8 IL-6, and TNF-α secretion of keratinocytes in vitro^28^. Besides, PMA-induced NETs promoted the secretion of LCN2, IL36G, and chemokines via activating TLR4 and NF-κB signaling pathways^29^. Our results also showed that ADSC-CM weakened the ability of NETs to induce inflammation. Different physiological conditions might affect the containing of NETs and their roles in infection and inflammation^30^. Hence, it is possible that ADSCs reduce the pro-inflammatory protein release of NETs.

However, there are still some limitations worth pondering in our study. Firstly, macrophages and sebaceous gland cells also play important roles in acne vulgaris. We only explored the effect of ADSCs on neutrophils and keratinocytes. The other important cellular types are warranted to be validated in acne vulgaris. Secondly, the protein and nucleic acid components of NETs are very complex. It is of great importance to determine the associated proteins or nucleic acids in participating *P. acnes*-related inflammation. Thirdly, our study only assessed NET levels at only one time point. The dynamic change of NET levels during acne vulgaris and the time of pathogenicity for NETs need further investigation. At last, it is just a preliminary preclinical study, the potential clinical application of ADSCs for treating acne vulgaris needs to be confirmed by more basic research and multicenter clinical trials.

## Conclusion

In conclusion, our results confirmed that NETs were enriched in the *P. acnes*-infected ear skin of mouse models, and could promote the proliferation, migration, and inflammatory cytokine secretion of keratinocytes in vitro. It is essential to clarify that the study conducted was an acute study, which implies that the observed effects may be limited to a short-term timeframe. The proliferation and migration of cells require longer observation in the future. More importantly, ADSCs could inhibit NET formation and reduce the effect of NETs to relieve acne vulgaris. Hence, our study preliminarily deciphered that ADSCs could attenuate *P. acnes*-related inflammation by inhibiting NET formation, thus providing a novel perspective of ADSCs in combating acne vulgaris.

### Supplementary Information

Supplementary Information.

## Data Availability

All the datasets displayed in this study can be obtained in the online database (https://www.jianguoyun.com/p/DZkC4WkQjNLdCxi_tI0FIAA). Further questions can be directed to the corresponding author.

## Abbreviations

EdU: 5-Ethynyl-20-deoxyuridine
ADSCs: Adipose-derived stem cells
BMN: Bone marrow neutrophils
BSA: Bovine serum albumin
CCK-8: Cell counting kit-8
CXCL1: Chemokine ligand 1
cit-H3: Citrullinated histone H3
CM: Conditioned medium
ECL: Enhanced chemiluminescenc
H&E: Hematoxylin–Eosin
HS: Hidradenitis suppurativa
IF: Immunofluorescence
IL-6: Interleukin 6
MPO: Myeloperoxidase
NETs: Neutrophil extrinsic traps
PAD-4: Peptidyl arginine deiminase 4
PBS: Phosphate-buffered saline
P. acnes: Propionibacterium acnes
PAPA: Pyoderma gangrenosum and acne
SDS-PAGE: Sodium dodecyl sulfate–polyacrylamide gel electrophoresis
