# Supplementary figures and images for: Neutrophil extracellular trap-related mechanisms in acne vulgaris inspire a novel treatment strategy with adipose-derived stem cells
Source: Sci Rep. 2024 Jan 17;14:1521. doi: 10.1038/s41598-024-51931-w (PMC10794178; doi:10.1038/s41598-024-51931-w)

Figure 3

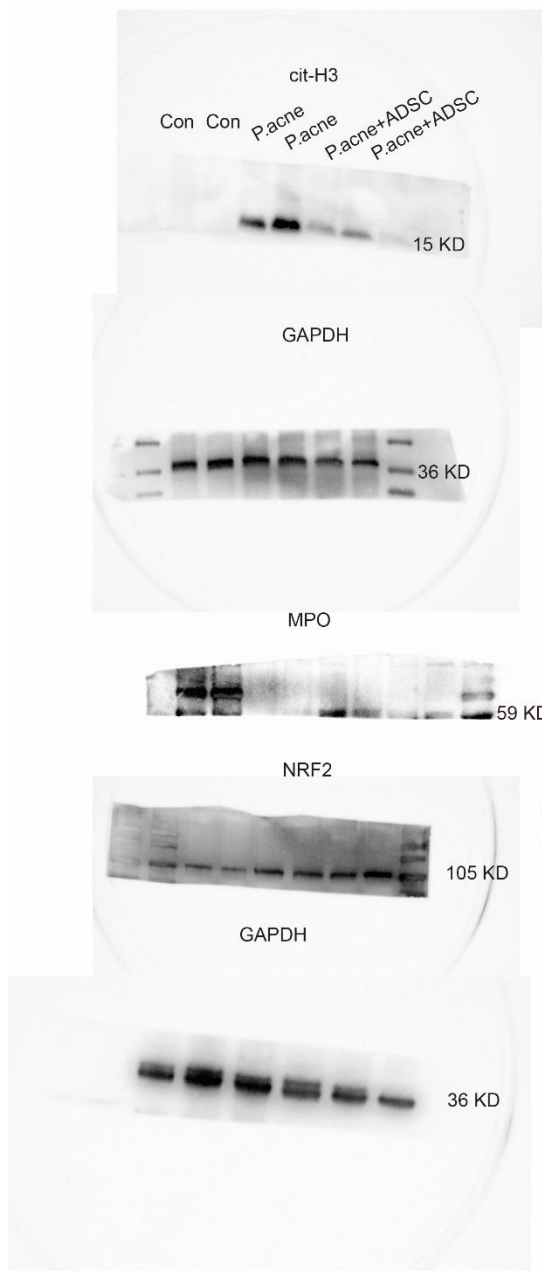

Figure 4

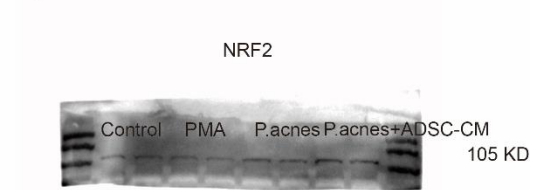

Figure 6

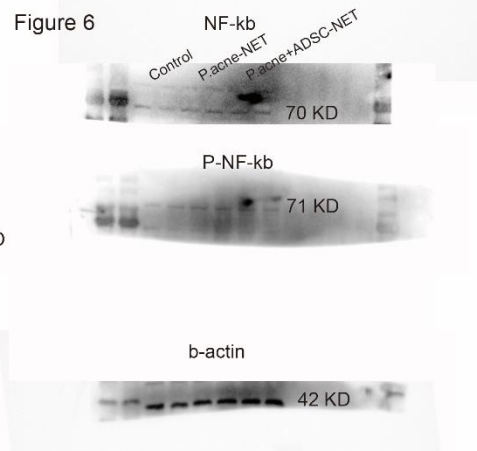

Supplement: Supplementary file 1 — Supplementary Information. [file 41598_2024_51931_MOESM1_ESM.pdf]
